# Availability of secondary healthcare data for conducting pharmacoepidemiology studies in Colombia: A systematic review

**DOI:** 10.1002/prp2.661

**Published:** 2020-09-23

**Authors:** Juan‐Sebastian Franco, David Vizcaya

**Affiliations:** ^1^ Medical Affairs Bayer S.A Bogotá Colombia; ^2^ Epidemiology Bayer Hispania Barcelona Spain

**Keywords:** Colombia, drug utilization, electronic health records, pharmacoepidemiology

## Abstract

Real‐world evidence (RWE) is emerging as a fundamental component of the post‐marketing evaluation of medicinal products. Even though the focus on RWE studies has increased in Colombia, the availability of secondary data sources to perform this type of research is not well documented. Thus, we aimed at identifying and characterizing secondary data sources available in Colombia. We performed a systematic literature review on PubMed, EMBASE, and VHL using a combination of controlled vocabulary and keywords for the concepts of electronic health records, epidemiologic studies and Colombia. A total of 323 publications were included. These comprised 123 identified secondary data sources including pharmacy dispensing databases, government datasets, disease registries, insurance databases, and electronic heath records, among others. These data sources were mostly used for cross‐sectional studies focused on disease epidemiology in a specific population. Almost all databases (95%) contained demographic information, followed by pharmacological treatment (44%) and diagnostic tests (39%). Even though the database owner was identifiable in 94%, access information was only available in 44% of the articles. Only a pharmacy‐dispensing database, local cancer registries, and government databases included a description regarding the quality of the information available. The diversity of databases identified shows that Colombia has a high potential to continue enhancing its RWE strategy. Greater efforts are required to improve data quality and accessibility. The linkage between databases will expand data pooling and integration to boost the translational potential of RWE.

## 1 INTRODUCTION

Real‐world evidence (RWE) is a fundamental component of the long‐term evaluation of medicinal products being increasingly requested by regulatory agencies, healthcare professional (HCPs) and payers to demonstrate the safe and appropriate use of these therapies, their effectiveness in every‐day treatment, and the cost‐effectiveness of the treatment strategies.^1^ RWE research using secondary data sources has multiple applications on therapeutics, including drug utilization studies,^2^, ^3^ post‐authorization safety studies,^4^ comparative effectiveness,^5^ and cost of interventions.^6^

Initiatives related with RWE across the world have highlighted its role as a valuable complement to the evidence generated in randomized controlled trials (RCTs).^7^ United States (US) and Europe have the highest number of healthcare‐related data sources, probably due to the structure of healthcare systems and the legal framework which facilitates the electronic collection of routine clinical care.^7^, ^8^ In Latin America, there is an increasing demand for effective and innovative treatments that drives the requirement for the continuous evaluation of their safety and effectiveness.^9^ A previous assessment of the status of RWE in Latin America has shown that, even though there are patient‐level data resources available, their quality varies and are locally managed without standardizing coding and practices.^10^

In Colombia, the situation is very similar. Even though there are government‐led information systems and a single‐payer healthcare system, the collection of data is not always complete.^10^, ^11^ The Colombian health system includes a social security system with public funding and a less preeminent private sector. The affiliation to a healthcare system is mandatory and is done through the health insurance companies (EPS—*entidades promotoras de la salud,* in Spanish*)* which manage the healthcare provision given by the healthcare settings. The goal of the Colombian healthcare system is to provide healthcare access to the entire of its population through a list of regimes that cover workers, low‐income population, and special populations such as armed forces.^12^, ^13^ Hence, the system management in Colombia is somewhat centralized although healthcare provision is scattered in various public and private entities. In order to improve and enhance the use of RWE in Colombia, it is important to have more information on the resources that could be used for such purposes. Hence, the objective of this study was to perform a systematic literature review to identify and describe the characteristics of the secondary healthcare data sources available in Colombia that have been used to date for conducting overall epidemiology and pharmacoepidemiology studies.

## 2 MATERIALS AND METHODS

### 2.1 Search strategy

A systematic literature review was carried out following the PRISMA guidelines.^14^ A comprehensive search strategy for peer‐reviewed articles was performed in three databases: PubMed, EMBASE, and Virtual Health Library, which includes Latin American sources. The search was conducted by the authors on December 2018. A combination of controlled vocabulary and keywords was used for the concepts of electronic health records (EHR), epidemiologic studies and Colombia; the Boolean operators “AND” and “OR” were used to combine these concepts (Supplementary Material 1). Given that, to the best of our knowledge, this is the first review of these characteristics conducted in Colombia, the search was not limited by publication date or language other than the date when we conducted the search: December 2018. All citations were imported into a citation management system and duplicates were removed.

### 2.2 Eligibility criteria

Analytical studies performed on secondary data sources originating from Colombia were considered for inclusion into the study; these include pharmacoepidemiological studies, pharmacoeconomic studies, and safety studies, among others. Secondary data sources were considered as articles that analyzed data already collected for other purposes, including healthcare records, administrative and commercial databases, and disease and drug registries. Finally, databases covering several countries and multi‐databases were also considered if they included information from Colombian patients.

The following articles were excluded from the review: studies performed under a “primary data collection” approach, defined as collection of data specifically for a particular study,^15^ studies that focused on single‐patient information (eg case reports, case series), pharmacoeconomic models, review articles, policy‐related articles, and studies not involving Colombian data.

An external qualified organization (PGA Farma) performed an initial screening of titles to select the eligible articles for abstract review and full‐text data extraction by the authors. Subsequently, the abstracts of all potentially relevant studies were reviewed by the authors to determine final inclusion.

### 2.3 Data extraction and analysis

Data were extracted using a standardized collection form, taking into account a template used in similar studies^2^, ^16^ and adapted from the methodological framework of the European Surveillance of Antimicrobial Consumption (ESAC) project^17^ and the Big Data for Better Outcomes project from the Innovative Medicines Initiatives.^18^

The format was structured into three parts. The first part described the general characteristics of the study, including main author, year of publication, type of publication (full article or poster), and whether additional countries were involved. The second section was relative to the study design, including variables such as type of study, purpose of the study, population included, and geographical level of analysis (ie national, regional or local). The third part addressed the variables related with the database, including data source (eg distributor, health insurance company, pharmacy, prescription, healthcare institution, government, and non‐government agencies), database design (ie administrative, commercial, EHR, disease registry, drug registry), database owner, health sector coverage, type of population coverage, therapeutic area according to the Medical Dictionary for Regulatory Activities (MedDRA) system‐organ classification (SOC),^19^ and patient‐level data information available.

The authors performed the data extraction on Microsoft Excel. In order to increase data quality, all the non‐free text cells were blocked for data entry to decrease the likelihood of entry errors and a subsequent quality assurance was performed by reviewing a sample of 50% of the extraction.

## 3 RESULTS

**FIGURE 1:**
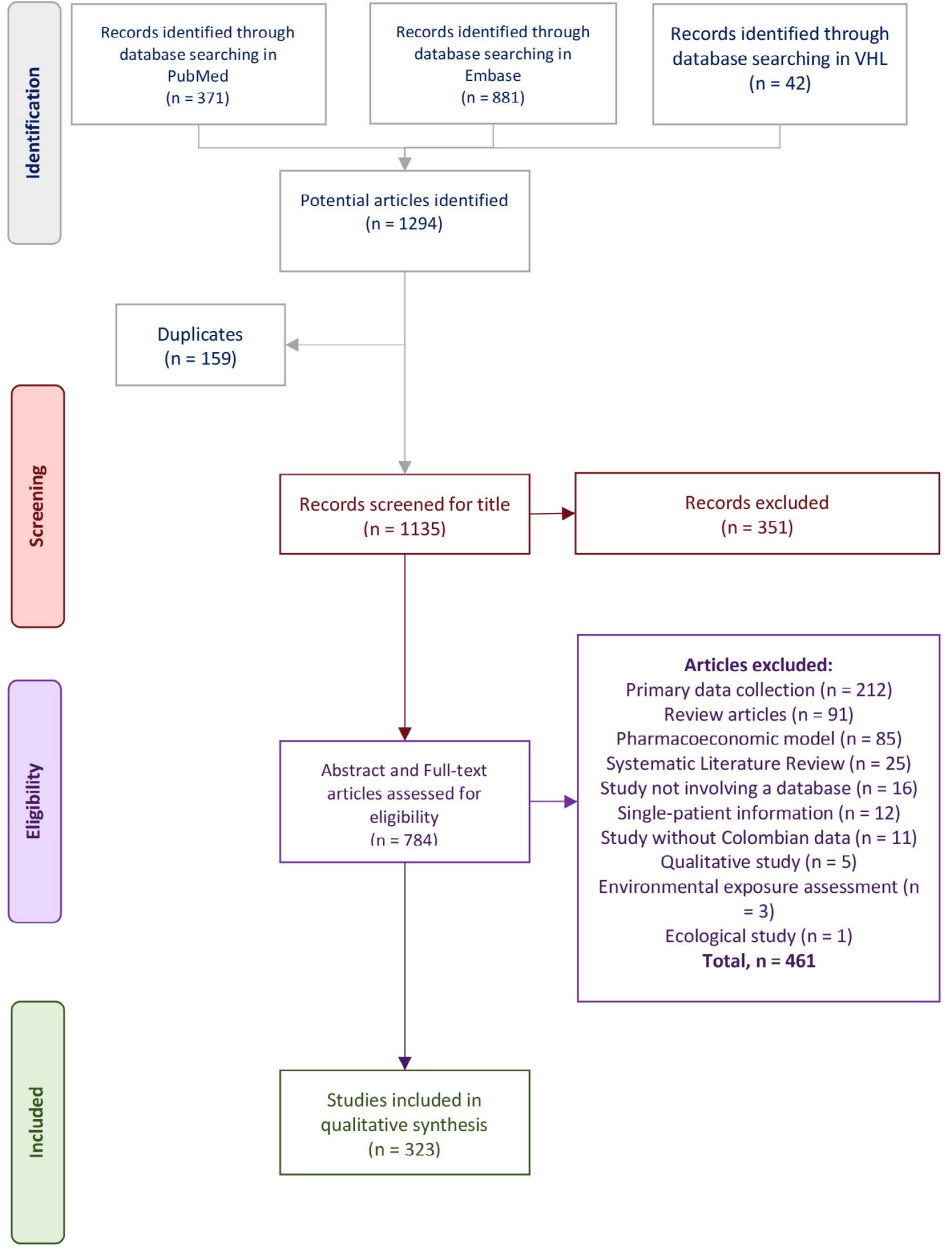
Flowchart of article selection. VHL: virtual health library

The PubMed, EMBASE, and VHL searches yielded a total of 1294 publications. Of these, 159 corresponded to duplicates, and thus, the titles of 1135 articles were screened. Among these, 351 articles were excluded after title screening and the abstract of the 784 remaining papers was assessed. Finally, 461 publications were excluded following the eligibility criteria and thus, 323 articles that had interpretable data and fulfilled the eligibility criteria were used for data extraction^20^, ^21^, ^22^, ^23^, ^24^, ^25^, ^26^, ^27^, ^28^, ^29^, ^30^, ^31^, ^32^, ^33^, ^34^, ^35^, ^36^, ^37^, ^38^, ^39^, ^40^, ^41^, ^42^, ^43^, ^44^, ^45^, ^46^, ^47^, ^48^, ^49^, ^50^, ^51^, ^52^, ^53^, ^54^, ^55^, ^56^, ^57^, ^58^, ^59^, ^60^, ^61^, ^62^, ^63^, ^64^, ^65^, ^66^, ^67^, ^68^, ^69^, ^70^, ^71^, ^72^, ^73^, ^74^, ^75^, ^76^, ^77^, ^78^, ^79^, ^80^, ^81^, ^82^, ^83^, ^84^, ^85^, ^86^, ^87^, ^88^, ^89^, ^90^, ^91^, ^92^, ^93^, ^94^, ^95^, ^96^, ^97^, ^98^, ^99^, ^100^, ^101^, ^102^, ^103^, ^104^, ^105^, ^106^, ^107^, ^108^, ^109^, ^110^, ^111^, ^112^, ^113^, ^114^, ^115^, ^116^, ^117^, ^118^, ^119^, ^120^, ^121^, ^122^, ^123^, ^124^, ^125^, ^126^, ^127^, ^128^, ^129^, ^130^, ^131^, ^132^, ^133^, ^134^, ^135^, ^136^, ^137^, ^138^, ^139^, ^140^, ^141^, ^142^, ^143^, ^144^, ^145^, ^146^, ^147^, ^148^, ^149^, ^150^, ^151^, ^152^, ^153^, ^154^, ^155^, ^156^, ^157^, ^158^, ^159^, ^160^, ^161^, ^162^, ^163^, ^164^, ^165^, ^166^, ^167^, ^168^, ^169^, ^170^, ^171^, ^172^, ^173^, ^174^, ^175^, ^176^, ^177^, ^178^, ^179^, ^180^, ^181^, ^182^, ^183^, ^184^, ^185^, ^186^, ^187^, ^188^, ^189^, ^190^, ^191^, ^192^, ^193^, ^194^, ^195^, ^196^, ^197^, ^198^, ^199^, ^200^, ^201^, ^202^, ^203^, ^204^, ^205^, ^206^, ^207^, ^208^, ^209^, ^210^, ^211^, ^212^, ^213^, ^214^, ^215^, ^216^, ^217^, ^218^, ^219^, ^220^, ^221^, ^222^, ^223^, ^224^, ^225^, ^226^, ^227^, ^228^, ^229^, ^230^, ^231^, ^232^, ^233^, ^234^, ^235^, ^236^, ^237^, ^238^, ^239^, ^240^, ^241^, ^242^, ^243^, ^244^, ^245^, ^246^, ^247^, ^248^, ^249^, ^250^, ^251^, ^252^, ^253^, ^254^, ^255^, ^256^, ^257^, ^258^, ^259^, ^260^, ^261^, ^262^, ^263^, ^264^, ^265^, ^266^, ^267^, ^268^, ^269^, ^270^, ^271^, ^272^, ^273^, ^274^, ^275^, ^276^, ^277^, ^278^, ^279^, ^280^, ^281^, ^282^, ^283^, ^284^, ^285^, ^286^, ^287^, ^288^, ^289^, ^290^, ^291^, ^292^, ^293^, ^294^, ^295^, ^296^, ^297^, ^298^, ^299^, ^300^, ^301^, ^302^, ^303^, ^304^, ^305^, ^306^, ^307^, ^308^, ^309^, ^310^, ^311^, ^312^, ^313^, ^314^, ^315^, ^316^, ^317^, ^318^, ^319^, ^320^, ^321^, ^322^, ^323^, ^324^, ^325^, ^326^, ^327^, ^328^, ^329^, ^330^, ^331^, ^332^, ^333^, ^334^, ^335^, ^336^, ^337^, ^338^, ^339^, ^340^, ^341^, ^342^ (Figure 1).

**FIGURE 2:**
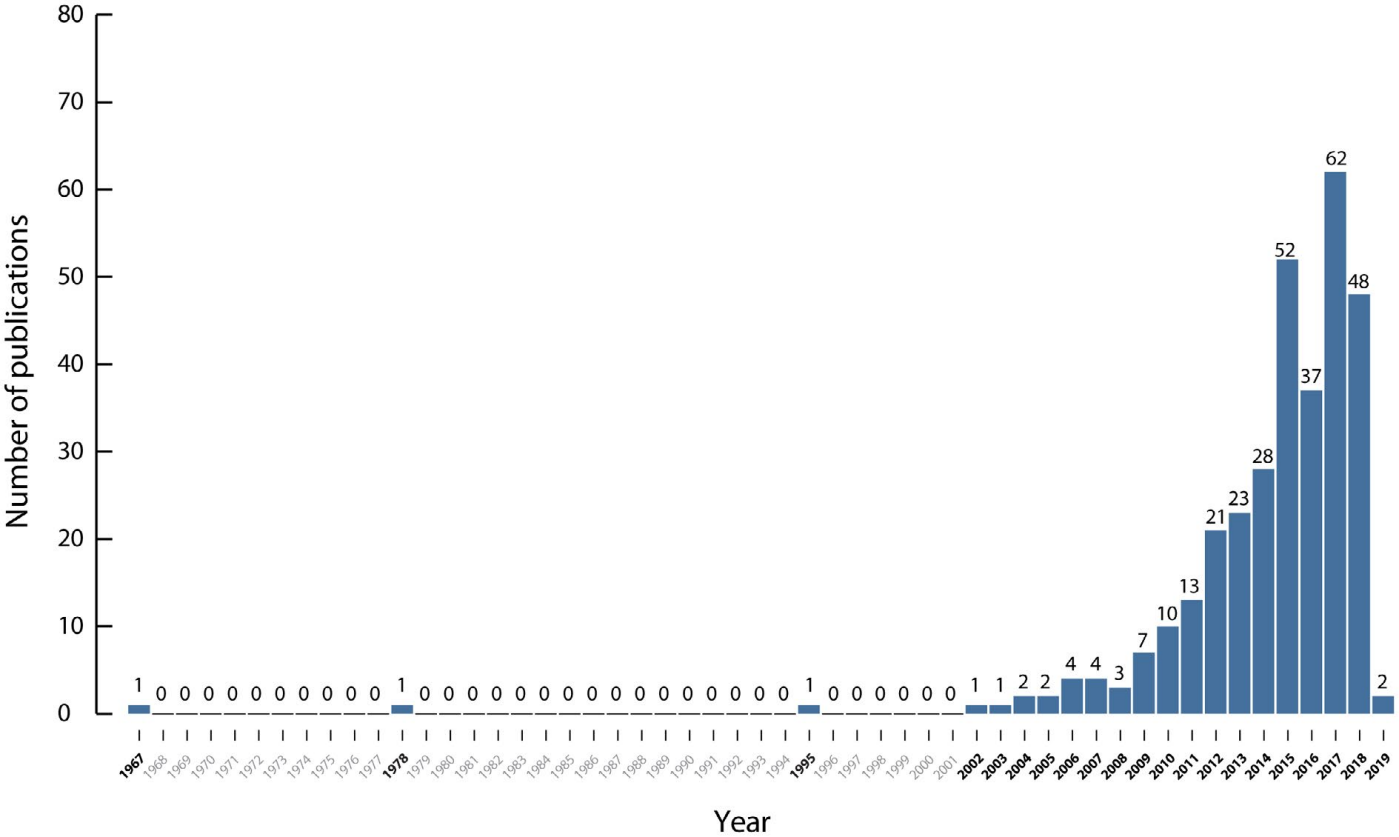
Number of publications per year

Until December 2018, 323 publications reported using secondary data sources to conduct epidemiology studies in Colombia.^26^, ^27^, ^28^, ^29^, ^30^, ^31^, ^32^, ^33^, ^34^, ^35^, ^36^, ^37^, ^38^, ^39^, ^40^, ^41^, ^42^, ^43^, ^44^, ^45^, ^46^, ^47^, ^48^, ^49^, ^50^, ^51^, ^52^, ^53^, ^54^, ^55^, ^56^, ^57^, ^58^, ^59^, ^60^, ^61^, ^62^, ^63^, ^64^, ^65^, ^66^, ^67^, ^68^, ^69^, ^70^, ^71^, ^72^, ^73^, ^74^, ^75^, ^76^, ^77^, ^78^, ^79^, ^80^, ^81^, ^82^, ^83^, ^84^, ^85^, ^86^, ^87^, ^88^, ^89^, ^90^, ^91^, ^92^, ^93^, ^94^, ^95^, ^96^, ^97^, ^98^, ^99^, ^100^, ^101^, ^102^, ^103^, ^104^, ^105^, ^106^, ^107^, ^108^, ^109^, ^110^, ^111^, ^112^, ^113^, ^114^, ^115^, ^116^, ^117^, ^118^, ^119^, ^120^, ^121^, ^122^, ^123^, ^124^, ^125^, ^126^, ^127^, ^128^, ^129^, ^130^, ^131^, ^132^, ^133^, ^134^, ^135^, ^136^, ^137^, ^138^, ^139^, ^140^, ^141^, ^142^, ^143^, ^144^, ^145^, ^146^, ^147^, ^148^, ^149^, ^150^, ^151^, ^152^, ^153^, ^154^, ^155^, ^156^, ^157^, ^158^, ^159^, ^160^, ^161^, ^162^, ^163^, ^164^, ^165^, ^166^, ^167^, ^168^, ^169^, ^170^, ^171^, ^172^, ^173^, ^174^, ^175^, ^176^, ^177^, ^178^, ^179^, ^180^, ^181^, ^182^, ^183^, ^184^, ^185^, ^186^, ^187^, ^188^, ^189^, ^190^, ^191^, ^192^, ^193^, ^194^, ^195^, ^196^, ^197^, ^198^, ^199^, ^200^, ^201^, ^202^, ^203^, ^204^, ^205^, ^206^, ^207^, ^208^, ^209^, ^210^, ^211^, ^212^, ^213^, ^214^, ^215^, ^216^, ^217^, ^218^, ^219^, ^220^, ^221^, ^222^, ^223^, ^224^, ^225^, ^226^, ^227^, ^228^, ^229^, ^230^, ^231^, ^232^, ^233^, ^234^, ^235^, ^236^, ^237^, ^238^, ^239^, ^240^, ^241^, ^242^, ^243^, ^244^, ^245^, ^246^, ^247^, ^248^, ^249^, ^250^, ^251^, ^252^, ^253^, ^254^, ^255^, ^256^, ^257^, ^258^, ^259^, ^260^, ^261^, ^262^, ^263^, ^264^, ^265^, ^266^, ^267^, ^268^, ^269^, ^270^, ^271^, ^272^, ^273^, ^274^, ^275^, ^276^, ^277^, ^278^, ^279^, ^280^, ^281^, ^282^, ^283^, ^284^, ^285^, ^286^, ^287^, ^288^, ^289^, ^290^, ^291^, ^292^, ^293^, ^294^, ^295^, ^296^, ^297^, ^298^, ^299^, ^300^, ^301^, ^302^, ^303^, ^304^, ^305^, ^306^, ^307^, ^308^, ^309^, ^310^, ^311^, ^312^, ^313^, ^314^, ^315^, ^316^, ^317^, ^318^, ^319^, ^320^, ^321^, ^322^, ^323^, ^324^, ^325^, ^326^, ^327^, ^328^, ^329^, ^330^, ^331^, ^332^, ^333^, ^334^, ^335^, ^336^, ^337^, ^338^, ^339^, ^340^, ^341^, ^342^, ^343^, ^344^, ^345^, ^346^, ^347^, ^348^ Since we had no limits set with regards to publication date, the first study identified in this SLR was published in 1967 and corresponds to a mortality study in children from Cali based on the death certificates of the local vital statistics office.^92^ The number of publications remained low until the 2010s, after which a total of 296 articles were published, corresponding to approximately 92% of the articles identified. Two articles were accepted for publication in 2018 but the journal published them in 2019 (Figure 2). Most publications were full‐text articles (71%), and 29% posters, mainly from scientific conferences. Only 34 articles (10%) included information of additional countries besides Colombia.^23^, ^50^, ^54^, ^85^, ^86^, ^159^, ^160^, ^169^, ^178^, ^181^, ^198^, ^206^, ^241^, ^260^, ^265^, ^267^, ^278^, ^279^, ^292^, ^304^, ^305^, ^307^, ^326^, ^331^, ^333^, ^334^, ^337^

The majority of the studies were cross‐sectional (61.6%), followed by cohort studies (11.5%). The main objective was often related with the description of disease epidemiology (70%), followed by drug utilization studies (20%). The proportion of drug effectiveness and safety studies was low, accounting for 3% and 6%, respectively.

A total of 123 databases were identified as the data source (Supplementary Material 2). The most frequently used database was from a pharmacy dispensing company, in 52 publications. This was followed by government databases (vital statistics in 29 publications and Ministry of Health datasets in 15). Finally, a local oncology disease registry was used in seven (7) publications. The main characteristics of the 123 databases identified are described in Table 1. The most common therapeutic areas were infections and neoplasms, both in 12%, followed by cardiac and neurologic disorders in 8% and endocrine disorders in 6% (Supplementary Material 3). Furthermore, even though the database owner was identifiable in 94%, access information and/or requirements were only available in 44% of the articles.

**TABLE 1:** Main characteristics of the databases identified

*Classification of data sources*	*Frequency*
Healthcare institution	40%
Government agency	27%
Pharmacy dispensing company	16%
Health insurance company	8%
Non‐government agency	4%
Prescription review	2%
Other	2%
Distributor	1%
*Database design*	*Frequency*
Electronic health records	30%
Administrative	35%
Disease registry	17%
Claims	11%
Survey	5%
Drug registry	1%
Commercial	1%
*Patient‐level data available*	*Frequency*
Patient demographics	95%
Pharmacological treatment	44%
Specific diagnostic tests	39%
Disease diagnosis by ICD‐10	38%
Adverse events	19%
Disease/Drug costs	12%
Drug codification by ATC	5%
Quality of Life (QoL)	1%

Abbreviations: ICD, International Classification of Diseases; ATC, Anatomical Therapeutic Chemical Classification System.

### 3.1 Global and regional data sources

A total of 18 databases with global or Latin American regional scope were identified (Table 2), including the World Health Organization's mortality database and the International Agency for Research on Cancer (IARC) report,^82^, ^159^, ^267^, ^278^, ^279^ as well as several Latin American disease registries and healthcare intervention data sets.

**TABLE 2:** Description of global and regional data sources

Data source name	Database summary	Population	Data type	Therapeutic Area (by MedDRA SOC)	Variables available in data source	Database owner/holder	Access possibility	Access requirements, including costs
International Agency for Research on Cancer (IARC)	Epidemiology of cancer and the study of potential carcinogens in the human environment	No Data	Disease specific	Neoplasms benign, malignant and unspecified (incl cysts and polyps)	Patient demographic information Disease diagnosis by ICD‐10	International Agency for Research on Cancer (IARC)	Yes	Free access through IARC's website
World Health Organization mortality database	Certified deaths for 20 selected cancer sites and total cancer mortality for the calendar period 1980‐2010	No Data	General Population	Neoplasms benign, malignant and unspecified (incl cysts and polyps)	Patient demographic information Disease diagnosis by ICD‐10	World Health Organization (WHO)	Yes	Accessible through WHO website
United Nations High Commissioner for Refugees (UNHCR)	Number of forcibly displaced persons (ie the sum of the number of refugees, IDPs, and asylum seekers)	No Data	General Population	Social circumstances	Patient demographic information	United Nations	Yes	UNHCR website
ACute Coronary Events—a multinational Survey of current management Strategies (ACCESS Registry)	Registry performed in developing countries that enrolled patients hospitalized with a diagnosis of Acute Coronary Syndrome	11,731	Disease specific	Cardiac disorders	Patient demographic information Specific diagnosis test Pharmacological treatment prescribed	ACCESS Registry investigators	No Data	No Data
Carotid body tumors (CBTs) database	All patients who underwent CBT resection in the years between 2004 and 2014 at 16 institutions	356	Disease specific	Neoplasms benign, malignant and unspecified (incl cysts and polyps)	Patient demographic information Disease diagnosis by ICD‐10 Pharmacological treatment prescribed	Article's authors	No Data	No Data
Hemato‐Oncology Latin America Observational Registry (HOLA)	Registry of selected hematological malignancies	Multiple Myeloma: 1,103 Chronic lymphocytic leukemia: 472 Non‐Hodgkin lymphoma: 1,975	Disease specific	Neoplasms benign, malignant and unspecified (incl cysts and polyps)	Patient demographic information Specific diagnosis test Pharmacological treatment prescribed	Hemato‐Oncology Latin America Observational Registry (HOLA)	No Data	No Data
QUALIDIAB database	QUALIDIAB is a program that evaluates the quality of care provided to people with diabetes in Latin America	3,099	Disease specific	Endocrine disorders	Patient demographic information Specific diagnosis tests Pharmacological treatment prescribed Quality of Life (QoL) information	Centro de Endocrinología Experimental y Aplicada (CENEXA)	No Data	No Data
Primary immunodeficiency (PIDs) Registry	Study prevalence and to promote awareness about Primary immunodeficiency (PIDs) in Mexico, and Central and South America	1,888	Disease specific	Immune system disorders	Patient demographic information Specific diagnosis test	Latin American Society for Immunodeficiencies (LASID)	No Data	No Data
Latin‐American Collaborative Study of Congenital Malformations (ECLAMC)	Epidemiological investigation of developmental congenital anomalies in Latin American hospital births	46,850	Disease specific	Pregnancy, puerperium and perinatal condition	Patient demographic information Specific diagnosis test	Latin‐American surveillance program (ECLAMC)	Yes	Information available at ECLAMC website (http://www.eclamc.org/eng/index.php)
Registry on the Management of Acute Diarrhea in Children (REMAD)	Infants/children (6 mo. to 6 y of age) presenting with community acquired acute diarrhea at onset	1,439	Disease specific	Infections and infestations	Patient demographic information Specific diagnosis test Pharmacological treatment prescribed	Registry on the Management of Acute Diarrhea in Children (REMAD)	No Data	No Data
Fresenius Medical Care Latin America database (EuCliD)	Database of hemodialysis patients	21,672	Disease specific	Renal and urinary disorders	Patient demographic information Specific diagnosis test	Fresenius Medical Care	No Data	No Data
Global Registry and Surveillance System for Diabetes (GRAND)	GRAND is a web‐based, centralized database designed to track individuals with diabetes and monitor their clinical information over time	1,742	Disease specific	Endocrine disorders	Patient demographic information Specific diagnosis test Pharmacological treatment prescribed	Global Registry and Surveillance System for Diabetes (GRAND)	No Data	No Data
Sistema de Redes de Vigilancia de los Agentes Responsables de Neumonias y Meningitis Bacterianas (SIREVA II)	Regional Vaccine System, which reports the isolated serotypes of S. pneumoniae, H. influenzae and N. meningitidis that causes bacterial pneumonia and meningitis	466	Disease specific	Infections and infestations	Patient demographic information Specific diagnosis test	Pan American Health Organization (PAHO)	Yes	Reports are available in PAHO website.
Pfizer International Growth Database. (KIGS)	Registry of patients under treatment with recombinant growth hormone (rhGH)	1,175	Disease specific	Endocrine disorders	Patient demographic information Specific diagnosis test Pharmacological treatment prescribed Adverse event information	Pfizer	No Data	No Data
Per‐oral endoscopic myotomy (POEM) registry	Patients with diagnosis of achalasia who underwent per‐oral endoscopic myotomy (POEM)	89	Disease specific	Gastrointestinal disorders	Patient demographic information Specific diagnosis test	Per‐oral endoscopic myotomy (POEM) registry	No Data	No Data
Latin‐American Catheter Ablation Registry	Analyze all ablations, either first time or re‐do procedures, performed in 2012	14,349	Disease specific	Cardiac disorders	Patient demographic information Specific diagnosis test	Latin American Society of Electrophysiology and Cardiac Stimulation (SOLAECE)	No Data	No Data
Product Surveillance Registry (PSR) for InterStim therapy	Patients under treatment with InterStim therapy for urinary incontinence, frequency and incomplete bladder emptying	910	Disease specific	Nervous system disorders	Patient demographic information	InterStim device owners	No Data	No Data
Medtronic Implantable Cardioverter Device (ICD) registry	Electronic database of patients with ICD in Latin America	42	Disease specific	Cardiac disorders	Patient demographic information	Medtronic	No Data	No Data
National databases	Direct and indirect costs of GI and CV events associated with common pain relievers for Rheumatoid Arthritis and Osteoarthritis patients	No Data	Disease specific	Musculoskeletal and connective tissue disorders	Disease/ Drug costs	No Data	No Data	No Data

Abbreviations: MedDRA: Medical Dictionary for Regulatory Activities; SOC: system‐organ classification; ICD‐10: International Statistical Classification of Diseases and Related Health Problems version 10; GI: gastrointestinal; CV: cardiovascular.

### 3.2 Government Databases

The government databases were secondary data sources commonly used in the articles identified. A total of 88 articles (27%) reported data from a government agency. The most frequently used data source was the vital statistics information, mainly on mortality, from the National Administrative Department of Statistics (DANE).^28^, ^30^, ^31^, ^38^, ^39^, ^45^, ^66^, ^69^, ^71^, ^74^, ^89^, ^93^, ^165^, ^171^, ^199^, ^200^, ^210^, ^221^, ^225^, ^228^, ^229^, ^252^, ^262^, ^268^, ^269^, ^274^, ^287^, ^303^, ^317^, ^321^, ^339^

The DANE collects the information through births and death certificates (classified using International Statistical Classification of Diseases and Related Health Problems version 10 (ICD‐10) codes, as well as through surveys and census of the population.^343^

SISPRO (Integrated Information System on Social Protection) integrates more than 10 primary sources of health‐related information in a single query system.^38^, ^39^, ^200^, ^266^, ^344^ Access to SISPRO requires a client access server, which allows the information to be on a local computer. Within this information system, the RIPS (Individual Registry of Health Services), contains data on age, gender, and medical diagnosis by ICD‐10 for patients treated by the health system (public and private providers).^64^, ^79^, ^87^, ^147^, ^154^, ^170^, ^197^, ^216^, ^217^, ^219^, ^229^, ^262^, ^275^, ^314^, ^338^

The High Cost Account (CAC), which was created by the government in 2007 and is administered by insurers, collects healthcare data on high‐cost diseases (eg cancer, diabetes, hemophilia, rheumatic diseases, etc).^21^, ^22^, ^132^, ^212^, ^234^, ^243^, ^248^, ^250^, ^251^, ^277^ This database has a quality control process (audit through a validation mesh and verification against the medical record) and contains demographic data, diagnosis by ICD‐10 and prescriptions.

### 3.3 Administrative and Claims Datasets

A pharmacy dispensing company (Audifarma SA) was identified in the SLR as the most commonly used database for RWE.^34^, ^42^, ^61^, ^63^, ^72^, ^100^, ^101^, ^102^, ^103^, ^104^, ^105^, ^106^, ^107^, ^108^, ^109^, ^111^, ^112^, ^113^, ^114^, ^115^, ^117^, ^118^, ^120^, ^122^, ^123^, ^124^, ^125^, ^126^, ^127^, ^128^, ^129^, ^130^, ^131^, ^134^, ^135^, ^136^, ^137^, ^138^, ^139^, ^140^, ^141^, ^163^, ^168^, ^242^, ^301^, ^310^ It contains demographic data, diagnosis by ICD‐10, and drug dispensation records coded by the Anatomical Therapeutic Chemical (ATC) classification, which can be used to identify comorbidities, drug‐to‐drug interactions, medication errors, adverse events, and calculate adherence rates. The company has its own pharmacoepidemiology department that performs quality control measures and the records are updated daily. The most common therapeutic areas explored with this database are cardiac (23%), neurological (21%) and endocrine (15%) disorders, with a focus on drug utilization (77%) and drug safety (17%) studies.

Regarding health insurance companies, a total of 27 articles reported the use of claims data as the source of data.^26^, ^27^, ^58^, ^78^, ^116^, ^140^, ^149^, ^175^, ^205^, ^226^, ^227^, ^231^, ^258^, ^270^, ^281^, ^284^, ^288^, ^289^, ^324^, ^325^ In most of the articles (75%), the specific health insurance company was not identifiable.

### 3.4 Local Disease and Drug Registries

There were 19 local disease and drug registries identified in 36 publications (Table 3). The most commonly used local registry was the population‐based cancer registry (PBCR) in Cali.^36^, ^214^, ^233^, ^280^, ^318^, ^320^, ^322^ The collection of the data is performed through active search and notification from hospitals, public and private laboratories, and the DANE. This database contains patient's demographic information and clinical diagnosis by ICD‐10, used to assess cancer morbidity and mortality. Additional PBCRs were created in Bucaramanga, Manizales, Pasto, and Barranquilla, with a coverage of 12% of the population.^166^, ^318^, ^319^ Furthermore, the Cali, Bucaramanga, Manizales, and Pasto PBCRs follow IARCs standards and are sources of their worldwide cancer incidence report.

**TABLE 3:** Description of local disease and drug registries

Data source name	Database Summary	Population	Data type	Therapeutic area (by MedDRA SOC)	Variables available in Data Source	Database owner/holder	Access possibility	Access requirements, including costs
Cali population‐based cancer registry (PBCR)	Cancer cases reports actively captured by linkage of different sources of information	9,804	Disease specific	Neoplasms benign, malignant and unspecified	Patient demographic information Disease diagnosis by ICD‐10	Cali population‐based cancer registry (PBCR)	No Data	No Data
Childhood Cancer Outcomes Surveillance System (VIGICANCER)	Patients younger than19 years of age, registered at any of the five Pediatric Oncology Units in Cali, with a new diagnosis of a malignant neoplasm	1,711	Disease specific	Neoplasms benign, malignant and unspecified	Patient demographic information Disease diagnosis by ICD‐10	Cali population‐based cancer registry (PBCR)	No Data	No Data
Bucaramanga Cancer Registry	The registry actively identifies, collects, and registers cancer cases by making regular visits to the primary information sources since 2000	1,039	Disease specific	Neoplasms benign, malignant and unspecified	Patient demographic information Disease diagnosis by ICD‐10	Bucaramanga Cancer Registry	No Data	No Data
Manizales Cancer Registry	Population‐based cancer registry in the city of Manizales up to IARC standards since 2003	1,482	Disease specific	Neoplasms benign, malignant and unspecified	Patient demographic information Disease diagnosis by ICD‐10	Manizales Cancer Registry	No Data	No Data
Pasto Cancer Registry	Population‐based cancer registry in the city of Pasto, with the inclusion of all malignant tumors diagnosed in individuals during the 1998‐2007 period	4,986	Disease specific	Neoplasms benign, malignant and unspecified	Patient demographic information Disease diagnosis by ICD‐10	Pasto Cancer Registry	No Data	No Data
Barranquilla Cancer Registry	Population‐based cancer registry in the city of Barranquilla, with the inclusion of all malignant tumors diagnosed in individuals since 2007	8,182	Disease specific	Neoplasms benign, malignant and unspecified	Patient demographic information Disease diagnosis by ICD‐10	Barranquilla Cancer Registry	No Data	No Data
Colombian Registry of Cardiovascular Disease (RECODEC)	Patients diagnosed with acute heart failure between 2011 and 2015	736	Disease specific	Cardiac disorders	Patient demographic information Disease diagnosis by ICD‐10	Fundación Santa Fe de Bogota (FSFB)	No Data	No Data
ROCI Registry	Patients with heart failure, treated according to the Optimize Colombia Program	436	Disease specific	Cardiac disorders	Patient demographic information Disease diagnosis by ICD‐10	ROCI Registry	No Data	No Data
Surveillance Program of Congenital Malformations	Registry of congenital malformations in newborns from Bogota and Cali	76,155	Disease specific	Congenital, familial and genetic disorders	Patient demographic information Specific diagnosis test	Programa de Vigilancia de Anomalías Congénitas	No Data	No Data
GenPE (Genetics and Pre‐eclampsia) Colombian registry	Primigravid women included at the time of delivery between December 2000 and February 2012.	2,028	Disease specific	Pregnancy, puerperium and perinatal condition	Patient demographic information Specific diagnosis test	GenPE (Genetics and Pre‐eclampsia) Colombian registry	Yes	Author information and/or website (www.genpe.org)
Bogota Congenital Malformations Surveillance Program (BCMSP)	Data on malformations from 2 sources: National Public Health Surveillance System (SIVIGILA), and electronic health records	431,670	Disease specific	Congenital, familial and genetic disorders	Patient demographic information Disease diagnosis by ICD‐10	Bogotá Congenital Malformations Surveillance Program (BCMSP)	No Data	No Data
Trauma Registry of the Pan American Society of Trauma	Contains information on trauma patients from two healthcare institutions: Hospital Universitario del Valle (HUV) and Fundacion Valle del Lili (FVL)	18,995	Disease specific	Injury, poisoning and procedural complications	Patient demographic information	Pan‐American Trauma Registry (PATR)	No Data	No Data
Grupo para el Control de la Resistencia Bacteriana de Bogotá (GREBO)	Microbiological surveillance network including 27 reference hospitals in the following Colombian cities	84,664	Disease specific	Infections and infestations	Specific diagnosis test	Grupo para el Control de la Resistencia Bacteriana de Bogotá (GREBO)	No Data	Contact information with the infectologists available at www.grupogrebo.org
Registry of Juvenile Patients with Polyautoimmunity	Disease registry of patients with polyautoimmunity documented at pediatric rheumatology clinics (17 centers on 5 Colombian cities)	313	Disease specific	Musculoskeletal and connective tissue disorders	Patient demographic information Specific diagnosis test	Pediatric Rheumatologists group	No Data	No Data
Nefrored	Multicenter registry of patients with confirmed diagnosis of lupus nephritis and primary glomerulonephritis through renal biopsy	763	Disease specific	Renal and urinary disorders	Patient demographic information Specific diagnosis test	NEFRORED	No Data	Project information in http://www.nefrored.org/
RedLANO (Latin American Neuro‐Oncology Network)	A bidirectional registry of glioblastoma patients treated in various institutions from Colombia throughout the last 5 years	213	Disease specific	Nervous system disorders	Patient demographic information Specific diagnosis test Pharmacological treatment prescribed	RedLANO (Latin American Neuro‐Oncology Network)	No Data	No Data
Colombian Health Care Workers (HCW) registry	HCWs who presented an episode of exposure to blood borne pathogens caused by percutaneous injuries or mucous membranes contamination	2,403	Disease specific	Infections and infestations	Patient demographic information Specific diagnosis test Pharmacological treatment prescribed	Servicios y Asesorías en Infectología (SAI)	No Data	No Data
Centro de Información Gestión e Investigación en Toxicología (CIGITOX)	Data related to accidents by poisonous animals, reported from all over the country by telephone to CIGITOX, and whose record was in its database	34,994	Disease specific	Injury, poisoning and procedural complications	Patient demographic information	Universidad Nacional de Colombia	Yes	No Data
Drug Eluting Stent (DREST) registry	Patients who underwent percutaneous coronary intervention for acute coronary event at Fundación Valle de Lili	3,056	Disease specific	Cardiac disorders	Patient demographic information Specific diagnosis test Pharmacological treatment prescribed	Fundacion Valle de Lili	No Data	No Data

Abbreviations: MedDRA, Medical Dictionary for Regulatory Activities; SOC, system‐organ classification; ICD‐10, International Statistical Classification of Diseases and Related Health Problems version 10.

In addition to the oncology databases, there were registries identified in diseases including heart failure (RECODEC^290^, ^291^ and ROCI^308^ registries), trauma,^180^, ^183^, ^184^, ^208^, ^309^ infectious diseases (GREBO^90^, ^311^, ^312^ healthcare workers occupational exposure registry^192^), and glomerulonephritis (Nefrored^174^, ^177^, ^188^), among others.^37^, ^146^, ^185^, ^187^, ^192^, ^220^, ^238^, ^255^, ^264^

### 3.5 Local single institution datasets

A total 84 articles included data from EHR of 49 healthcare institutions around Colombia. In 41 articles (84%), the healthcare institution was identifiable and included a mix of public and private institutions. The healthcare institutions with the greater amount of publications are located in Colombia's three major cities: Bogota (Hospital Universitario San Ignacio^57^, ^91^, ^176^, ^247^ and Instituto Nacional de Cancerologia^43^, ^158^, ^195^, ^313^), Medellin (Hospital Pablo Tobon Uribe^172^, ^201^, ^261^, ^297^, ^328^) and Cali (Fundacion Valle de Lili^60^, ^196^, ^330^, ^335^, ^336^, ^340^).

### 3.6 Commercial and Miscellaneous Databases

IMS health was among the commercial databases identified in the SLR with a total of two (2) publications involving Colombian patient's data^206^, ^260^ on drug utilization using retail prescription sales data. Among the miscellaneous data sources (ie databases not fitting any of the earlier sections), we found the Healing the Children (HTC) organization database that include data on patients with cleft disease,^143^; a database on genetic diseases^29^; and a database on primary central nervous system tumors from pathology reports.^213^

## 4 DISCUSSION

Secondary data sources correspond to a rich source of information that have become extensively used over the recent years.^345^, ^346^ This SLR highlights the main characteristics of the 123 databases used for secondary research in Colombia identified in 323 articles. The databases identified constitute a mix of administrative data, EHR and registries originating from government agencies, healthcare institutions and pharmacy dispensing companies. These data sources were particularly used for cross‐sectional studies focused on portraying disease epidemiology in a particular population.

Colombia has a long‐standing tradition of systematically collecting data from different sources and utilize it to perform epidemiologic research, as shown in this review. Moreover the country has been keeping up with global and regional trends on capturing and using secondary data sources since the first publication using local vital statistics was in 1967 and Cali's PBCR was set‐up in 1962,^36^, ^92^ similar to global (IARC was created in 1965) and regional databases (ECLAMC on congenital malformations was established in 1967). A recent analysis of RWE in Latin America published by Justo et al, shows that the landscape of Colombia is similar to Chile, with well‐established national systems and registries, and ahead of Argentina, where there's still fragmented data source management and sporadic use of RWE data for decision‐making.^10^ Moreover the structure of Colombian health system including but not limited to the structure of EPS groups, government owned electronic infrastructures such as the High‐Cost Account, electronic registers availability, and a pressing demand for more evidence‐based decision‐making among regulators, HTA and HMO bodies, and drug manufacturers or distributors should facilitate future developments in RWE in the near future. The fact that only a small part of the interactions with the health system happens outside of the general system of social security in health (*SGSSS—Sistema General de Seguridad Social en* *S* *alud*, in Spanish) makes it even more appealing to further explore the capabilities of using secondary data sources from Colombian structures related to health.^13^

A single pharmacy dispensing company (Audifarma SA) was the most frequently used source of secondary data, corresponding to 16% of the total articles identified.^34^, ^42^, ^61^, ^63^, ^72^, ^100^, ^101^, ^102^, ^103^, ^104^, ^105^, ^106^, ^107^, ^108^, ^109^, ^111^, ^112^, ^113^, ^114^, ^115^, ^117^, ^118^, ^120^, ^122^, ^123^, ^124^, ^125^, ^126^, ^127^, ^128^, ^129^, ^130^, ^131^, ^134^, ^135^, ^136^, ^137^, ^138^, ^139^, ^140^, ^141^, ^163^, ^168^, ^242^, ^301^, ^310^ This is the largest pharmacy dispensing company in Colombia (ie logistics operator), covering approximately 6.5 million affiliates that corresponds to 13.2% of the country's population. The sample size and the consistent use of identifiers (eg ICD‐10, ATC) are the main advantages that allows to study drug utilization patterns and even drug safety in routine clinical practice. Its major disadvantage is the current unavailability of linkage with clinical data to create more robust datasets to study specific health and drug outcomes.

Government databases were also commonly used as secondary data sources. For many decades, the country has been implementing information systems that allows the government to capture diverse health‐related data at a population level.^229^ The access to these databases is readily available and includes a great amount of patient‐level data.^347^ Taking into account that in Colombia the access to healthcare is universal, the entire population is covered by the data collection and non‐participation should be minimal.^266^ However, those without access to care or who failed to encounter the health system (eg geographical location) will not be captured. Additionally, being a passive reporting system, these databases relies on the appropriate reporting behaviors and thus, there is a risk for underreporting.^322^, ^348^

In contrast to passive government databases, disease registries are based on active finding of all new cases of disease from a well‐defined demographic area, which could improve the data reliability.^322^ In Colombia, several local disease registries were identified, mainly in oncology.^36^, ^318^ While these registries currently focus on cancer incidence and mortality, it would be ideal if in the future they include data on cancer treatment to expand the scope of the endpoints analyzed.

The main challenge with secondary data sources is that many of the available data currently rely in separate silos.^349^ The absence of shared identifiers between the different types of databases prevents information linkage among heterogeneous data sources,^346^, ^349^ which can be attributable not only to technical difficulties, but also to privacy concerns.^346^, ^349^ Moreover interpretations of results coming from studies using secondary data sources will continue to encounter distrust, mainly due to data quality that could incorporate bias originating from confounding, missing data and misclassification.^345^, ^346^, ^349^ A knowledge gap related to data quality was identified in this study, since only a pharmacy dispensing company, the PBCRs and the government databases included a description of quality control measures or of the overall quality of the information available.

The present SLR had some limitations that need to be accounted for when interpreting its results. First, the analyses were made at the article‐level and not the database‐level, given the methodology of the SLR. This means that only the data sources that have been used for publication purposes were included in the SLR and there can be other data sources not reported in the literature. Furthermore, the same sources produced several articles, leading to a potential over‐representation of these databases originated from research groups with high productivity in terms of publications. This also assures that databases with enough quality for epidemiological research had higher chances of being detected. Second, some details on the databases used in some of these studies are not available in the published article but only available on websites. This depended mainly on the author's description of the data source or on the specificities of each database, which may not be extensively described in each study. Finally, we reported an increase in the number of publications during the last decade, which could be because the search strategy was not able to capture earlier studies that did not properly described the data source used, which could lead to an underrepresentation of some databases. Nonetheless, this could also be a representation of an actual increase in research productivity using secondary data sources, following the trend observed in other relevant countries (eg US).

## 5 CONCLUSIONS

With the increasing access and use of these data sources, it is crucial that the evidence generated is made publicly available and that access to the data is granted to a larger research community, to the extent that this is possible, and assured through governance processes and ethics standards. A greater focus on expanding the use of these databases is required to increase their visibility in order to boost the translational potential of RWE in Colombia. The governance process to access most of the databases identified was poorly described or not described at all. Moreover replicating this SLR in other Latin American countries would contribute to the exploration of the status‐quo of RWE in the region that is required to further define and describe the databases available for pharmacoepidemiology research.

## CONFLICT OF INTEREST

Juan‐Sebastian Franco and David Vizcaya are full‐time employees of Bayer Colombia and Hispania (Spain), respectively.

## AUTHOR CONTRIBUTIONS

All authors conceptualized, designed the study, analyzed the data, interpreted the data, reviewed and revised the manuscript. Dr Franco drafted the initial manuscript. All authors approved the final manuscript as submitted and agree to be accountable for all aspects of the work.

## ETHIC STATEMENT

The authors state that no ethical approval was needed.

## Supporting information

Supplementary Material

## Data Availability

Research data are not available for sharing.
