# Supplementary material for: Availability of secondary healthcare data for conducting pharmacoepidemiology studies in Colombia: A systematic review
Source: Pharmacol Res Perspect. 2020 Sep 23;8(5):e00661. doi: 10.1002/prp2.661 (PMC7510335; doi:10.1002/prp2.661)
Supplement: Supplementary file 1 — Supplementary Material [file PRP2-8-e00661-s001.docx]

**Supplementary Materials**

**Supplementary Material 1: Search Notes**

**I. Research question**

What are the characteristics of secondary data sources used for RWE studies in Colombia?

**II. Main concepts**

Electronic health records AND epidemiologic studies AND Colombia

Electronic health records: Topic specific query in PubMed (<https://www-nlm-nih-gov.ezp.welch.jhmi.edu/bsd/special_queries.html)>

Database: PubMed

| Search | Concept | Query | Items found |
| --- | --- | --- | --- |
| #1 | Electronic health records | "Electronic Health Records"[Mesh] OR "Databases, Factual"[Mesh] OR "Databases, Pharmaceutical"[Mesh] OR "Insurance Claim Review"[Mesh] OR "Drug Utilization"[Mesh] OR "Prescriptions"[Mesh] OR "Registries"[Mesh] | 264,548 |
| #2 | Electronic health records | “Electronic health records”[tw] OR health record*[tw] OR medical record*[tw] OR computerized medical record*[tw] OR database*[tw] OR “administrative database”[tw] OR administrative data*[tw] OR claims data*[tw] OR drug utilization*[tw] OR prescription*[tw] OR “drug prescription”[tw] OR registr*[tw] OR “patient registry”[tw] OR “drug registry”[tw] OR “population registry”[tw] | 917,659 |
| #3 | Electronic health records | #1 OR #2 | 933,469 |
| #4 | Epidemiologic studies | "Epidemiologic Studies"[Mesh] OR "Pharmacoepidemiology"[Mesh] OR "Economics, Medical"[Mesh] OR "Health Care Economics and Organizations"[Mesh] OR "Health Expenditures"[Mesh] | 3,579,394 |
| #5 | Epidemiologic studies | Epidemiological stud*[tw] OR observational stud*[tw] OR cohort stud*[tw] OR cross-sectional stud*[tw] OR case-control stud*[tw] OR retrospective stud*[tw] OR prospective stud*[tw] OR “non-interventional study”[tw] OR “healthcare economics”[tw] OR “health expenditure”[tw] OR pharmacoeconomic stud*[tw] | 2,077,864 |
| #6 | Epidemiologic studies | #4 OR #5 | 3,856,241 |
| #7 | Colombia | "Colombia"[Mesh] | 8,989 |
| #8 | Colombia | “Colombia”[tw] OR Colombia*[tw] | 14,174 |
| #9 | Colombia | #7 OR #8 | 14,174 |
| #10 | All | #3 AND #6 AND #9 | 371 |

Database: Embase

| Search | Concept | Query | Items found |
| --- | --- | --- | --- |
| #1 | Electronic health records | 'electronic health record'/exp OR 'data base'/exp OR 'drug database'/exp OR 'health insurance'/exp OR 'drug utilization'/exp OR 'prescription'/exp OR 'registration'/exp | 943,546 |
| #2 | Electronic health records | (‘Electronic health records’ OR health record* OR medical record* OR computerized medical record* OR database* OR ‘administrative database’ OR administrative data* OR claims data* OR drug utilization* OR prescription* OR ‘drug prescription’ OR registr* OR ‘patient registry’ OR ‘drug registry’ OR ‘population registry’):ab,ti | 448,246 |
| #3 | Electronic health records | #1 OR #2 | 1,143,492 |
| #4 | Epidemiologic studies | 'observational study'/exp OR 'pharmacoepidemiology'/exp OR 'health economics'/exp OR 'pharmacoeconomics'/exp | 950,664 |
| #5 | Epidemiologic studies | (Epidemiological stud* OR observational stud* OR cohort stud* OR cross-sectional stud* OR case-control stud* OR retrospective stud* OR prospective stud* OR ‘non-interventional study’ OR ‘healthcare economics’ OR ‘health expenditure’ OR pharmacoeconomic stud*):ab,ti | 2,107,242 |
| #6 | Epidemiologic studies | #4 OR #5 | 2,853,301 |
| #7 | Colombia | 'Colombia'/exp | 16,810 |
| #8 | Colombia | (‘Colombia’ OR Colombia*):ab,ti | 17,385 |
| #9 | Colombia | #7 OR #8 | 22,121 |
| #10 | All | #3 AND #6 AND #9 | 881 |

Database: Virtual Health Library (VHL)

| Search | Concept | Query | Items found |
| --- | --- | --- | --- |
| #1 | Electronic health records | (MH:"Electronic Health Records" OR MH:"Databases, Factual" OR MH:"Databases, Pharmaceutical" OR MH:"Insurance Claim Review" OR MH:"Drug Utilization" OR MH:"Prescriptions" OR MH:"Registries") | 174,860 |
| #2 | Electronic health records | (TW:(“Electronic health records” OR health record* OR medical record* OR computerized medical record* OR database* OR “administrative database” OR administrative data* OR claims data* OR drug utilization* OR prescription* OR “drug prescription” OR registr* OR “patient registry” OR “drug registry” OR “population registry”)) | 1,280 |
| #3 | Electronic health records | #1 OR #2 | 175,206 |
| #4 | Epidemiologic studies | (MH:"Epidemiologic Studies" OR MH:"Pharmacoepidemiology" OR MH:"Economics, Medical" OR MH:"Health Care Economics and Organizations" OR MH:"Health Expenditures") | 37,171 |
| #5 | Epidemiologic studies | (TW:(Epidemiological stud* OR observational stud* OR cohort stud* OR cross-sectional stud* OR case-control stud* OR retrospective stud* OR prospective stud* OR “non-interventional study” OR “healthcare economics” OR “health expenditure” OR pharmacoeconomic stud*)) | 168,142 |
| #6 | Epidemiologic studies | #4 OR #5 | 195,970 |
| #7 | Colombia | (MH:"Colombia") | 18,900 |
| #8 | Colombia | (TW:(“Colombia” OR Colombia*)) | 49,304 |
| #9 | Colombia | #7 OR #8 | 49.304 |
| #10 | All | #3 AND #6 AND #9 | 42 |

**III. Any limits**

None.

**IV. Any known records to include in search results**

Machado-Alba J, Fernández A, Castrillón JD, Campo CF, Echeverri LF, Gaviria A, Londoño MJ, Ochoa SA, Ruíz JO. Prescribing patterns and economic costs of proton pump inhibitors in Colombia. Colomb Med (Cali). 2013 Mar 30;44(1):13-8. PMID: 24892316

Machado-Alba JE, Calvo-Torres LF, García-Betancur S, Aguirre-Novoa A, Bañol-Giraldo AM. Drug utilisation study in patients receiving antiepileptic drugs in Colombia. Neurologia. 2016 Mar;31(2):89-96. PMID: 26304659

García LS, Bravo LE, Collazos P, Ramírez O, Carrascal E, Nuñez M, Portilla N, Millan E. Cali cancer registry methods. Colomb Med (Cali). 2018 Mar 30;49(1):109-120. PMID: 29983471

**Supplementary Material 2: Databases used for pharmacoepidemiology studies**

| **Data Source Name** | **Number of Articles** |
| --- | --- |
| Audifarma S.A. | 52 |
| National Administrative Department of Statistics (DANE) | 29 |
| Health Insurance Company  (nonspecific) | 20 |
| Individual Registry of Healthcare Provision (RIPS) | 15 |
| High Cost Account (CAC) | 10 |
| National System for Public Health Surveillance (SIVIGILA) | 8 |
| Healthcare institution (nonspecific) | 8 |
| Cali population-based cancer registry (PBCR) | 7 |
| Fundacion Valle de Lili | 6 |
| Hospital Pablo Tobon Uribe | 5 |
| International Agency for Research on Cancer (IARC) | 5 |
| Trauma Registry of the Pan American Society of Trauma | 5 |
| Childhood Cancer Outcomes Surveillance System (VIGICANCER) | 4 |
| Hospital Universitario San Ignacio | 4 |
| Instituto Nacional de Cancerología (INC) | 4 |
| Integrated Information System on Social Protection (SISPRO) | 4 |
| National Demographic Health Surveys (NDHS) | 4 |
| Biomab | 3 |
| Fundación Oftalmológica de Santander-FOSCAL | 3 |
| Grupo para el Control de la Resistencia Bacteriana de Bogotá (GREBO) | 3 |
| Hospital Universitario del Valle | 3 |
| Nefrored | 3 |
| Renal Therapy Services (RTS) | 3 |
| Salud Total EPS | 3 |
| Sistema de Información de Precios de Medicamentos (SISMED) | 3 |
| Bucaramanga Cancer Registry | 2 |
| Clinica El Bosque | 2 |
| Clinical History Administration System (SAHICO) | 2 |
| Colombian Registry of Cardiovascular Disease (RECODEC) | 2 |
| Dinamica IPS | 2 |
| Fundación Cardioinfantil | 2 |
| Hemato-Oncology Latin America Observational Registry (HOLA) | 2 |
| Hospital Universitario Hernando Moncaleano Perdomo de Neiva | 2 |
| Hospital Universitario San Jorge (Pereira) | 2 |
| Hospital Universitario San Vicente Fundación | 2 |
| IMS Health | 2 |
| Latin-American Collaborative Study of Congenital Malformations (ECLAMC) | 2 |
| Manizales Cancer Registry | 2 |
| Medicarte IPS | 2 |
| Organización Sanitas Internacional | 2 |
| QUALIDIAB database | 2 |
| Red Nacional de Donación y Trasplantes | 2 |
| RedLANO (Latin American Neuro-Oncology Network) | 2 |
| Secretaria Departamental de Salud del Valle | 2 |
| Sistema de Redes de Vigilancia de los Agentes Responsables de Neumonias y Meningitis Bacterianas (SIREVA II) | 2 |
| World Health Organization mortality database | 2 |
| ACute Coronary Events—a multinational Survey of current management Strategies (ACCESS Registry) | 1 |
| Barranquilla Cancer Registry | 1 |
| Bogota Congenital Malformations Surveillance Program (BCMSP) | 1 |
| Bogota's Pharmacovigilance Program | 1 |
| Carotid body tumors (CBTs) database | 1 |
| Centro de Información Gestión e Investigación en Toxicología (CIGITOX) | 1 |
| Centro Médico Palermo | 1 |
| Clinica CardioVid | 1 |
| Clinica de Marly | 1 |
| Clinica Maternidad Rafael Calvo C | 1 |
| Clínica Universitaria Bolivariana | 1 |
| Colombian Health Care Workers (HCW) registry | 1 |
| Colombian Red Cross | 1 |
| Colsubsidio | 1 |
| Corpus and Rostrum Plastic Surgery Clinic | 1 |
| Department of Health in Cordoba, Healthcare institution (nonspecific) | 1 |
| Departmet of Health in Caqueta | 1 |
| Drug Eluting Stent (DREST) registry | 1 |
| Fresenius Medical Care Latin America database (EuCliD) | 1 |
| Fundacion Cardiovascular de Colombia | 1 |
| Fundacion Colombiana de Cancerologia Clinica Vida | 1 |
| Fundación Oftalmológica Nacional (FUNDONAL) | 1 |
| Fundación Santa Fe de Bogota (FSFB) | 1 |
| Fundación Universitaria Del Área Andina | 1 |
| GenPE (Genetics and Pre-eclampsia) Colombian registry | 1 |
| Global Registry and Surveillance System for Diabetes (GRAND) | 1 |
| Group of primary immunodeficiencies | 1 |
| Healing the Children (HTC) organization cleft disease database | 1 |
| Higuera-Escalante Blood Bank | 1 |
| Hospital Antonio Roldán Betancur | 1 |
| Hospital de La Victoria, Secretaría Distrital de Salud Hospital El Salvador, Instituto de Genética Humana, Asociación Colombiana de Pacientes con Enfermedades de Depósito Lisosomal | 1 |
| Hospital de San Jose | 1 |
| Hospital Departamental de Villavicencio | 1 |
| Hospital La María de Medellín | 1 |
| Hospital Militar Central | 1 |
| Hospital Simon Bolivar | 1 |
| Hospital Universitario de Colombia | 1 |
| Hospital Universitario de Neiva | 1 |
| Hospital Universitario Infantil de San José | 1 |
| Hospital Universitario Mayor Méderi | 1 |
| Hypertensive patients database | 1 |
| Instituto Colombiano de Seguro Social | 1 |
| Instituto de Cancerología (IDC)-Clínica Las Américas | 1 |
| Instituto de Ortopedia Infantil Roosevelt | 1 |
| Latin-American Catheter Ablation Registry | 1 |
| Laura Daniela Clinic | 1 |
| Medtronic Implanatable Cardioverter Device (ICD) registry | 1 |
| Municipal Statistical Office | 1 |
| Mutual SER EPSS | 1 |
| National Administrative Registry of Cancer (RANC) | 1 |
| National databases | 1 |
| National Institute of Legal Medicine and Forensic Sciences | 1 |
| National Mental Health Survey | 1 |
| National Registry of Victims (RUV) | 1 |
| National Survey of Substance abuse | 1 |
| National Survey of the Nutritional Situation in Colombia (ENSIN) | 1 |
| Pasto Cancer Registry | 1 |
| Pathology Laboratory - Universidad de Antioquia | 1 |
| Per-oral endoscopic myotomy (POEM) registry | 1 |
| Pfizer International Growth Database. (KIGS) | 1 |
| Primary central nervous system tumours | 1 |
| Primary immunodeficiencies (PIDs) Registry | 1 |
| Product Surveillance Registry (PSR) for InterStim therapy | 1 |
| Programa de Salud Visual | 1 |
| Programa de Vigilancia de Anomalías Congénitas | 1 |
| Psychosocial Care Program and Victim Integral Health (PAPSIVI) | 1 |
| Quality of Life Survey in Medellin | 1 |
| Red Nacional de Bancos de Sangre y Servicios de Transfusion | 1 |
| Registry of Juvenile Patients with Polyautoimmunity | 1 |
| Registry on the Management of Acute Diarrhea in Children (REMAD) | 1 |
| Riesgo de fractura S.A (CAYRE) | 1 |
| ROCI Registry | 1 |
| SaludCoop EPS | 1 |
| Secretaria Distrital de Salud de Bogota | 1 |
| Sistema de vigilancia epidemiológica de violencia intrafamiliar y sexual (SIVIF) | 1 |
| Unidad de Gastroenterología, Hepatología y Nutrición Pediátrica (Gastronutriped) | 1 |
| United Nations High Commissioner for Refugees (UNHCR) | 1 |

**Supplementary Material 3: Therapeutic area of databases**

**
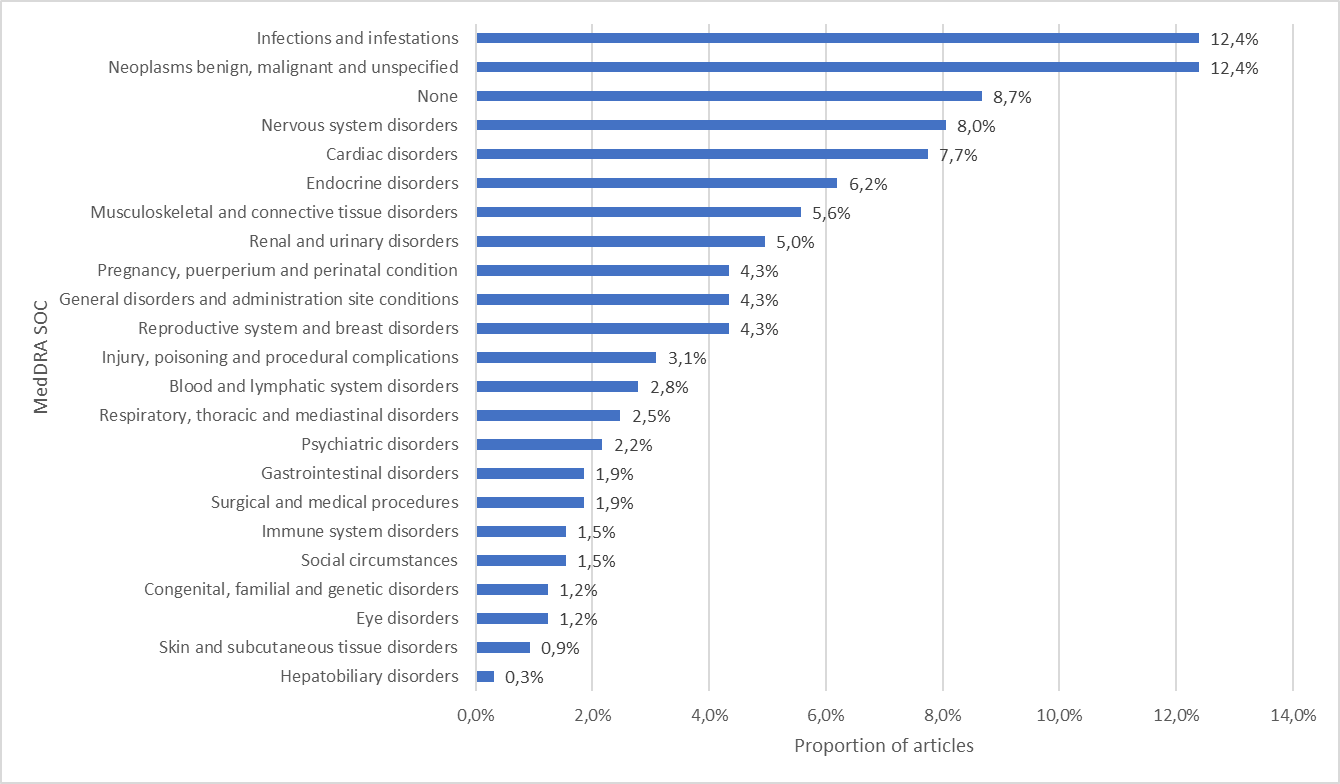
**

MedDRA: Medical Dictionary for Regulatory Activities; SOC: system-organ classification
